# Polymorphic self-assembly of pyrazine-based tectons at the solution–solid interface

**DOI:** 10.3762/bjnano.10.50

**Published:** 2019-02-18

**Authors:** Achintya Jana, Puneet Mishra, Neeladri Das

**Affiliations:** 1 Indian Institute of Technology Patna, Bihta, Patna-801106, India; 2 Central University of South Bihar, Gaya-824236, India

**Keywords:** highly oriented pyrolytic graphite (HOPG), organic molecules, pyrazine, pyridines, scanning tunneling microscopy, self-assembly

## Abstract

Exploring the surface self-assembly of small molecules that act as building blocks (tectons) for complex supramolecular structures is crucial for realizing surface-supported functional molecular devices. Here, we report on the synthesis and surface self-assembly of a new pyrazine-derived molecule with pyridine pendants. Ambient scanning tunneling microscopy investigation at the solution–solid interface reveals polymorphic self-assembly of these molecules on a HOPG substrate. Two different molecular packing structures with equal distribution are observed. Detailed analysis of the STM images emphasizes the crucial role of weak intermolecular hydrogen bonding, and molecule–substrate interactions in the formation of the observed polymorphs. Such weak hydrogen bonding interactions are highly desirable for the formation of modular supramolecular architectures since they can provide sufficiently robust molecular structures and also facilitate error correction.

## Introduction

Molecular materials are attracting considerable attention for the fabrication of next-generation functional devices owing to their high density and low power requirements. Several recent studies have highlighted the immense potential of molecules as a component of functional electronics [1–2], spintronics [3–5] and mechanical [6] devices. However, most of these studies employed extreme experimental conditions such as ultra-low temperatures, and ultra-high vacuum, which are far from any practically realizable application. On the other hand, exploring molecular functionalities under ambient conditions on technologically relevant two-dimensional surfaces such as graphene or MoS_2_ is highly desirable for realizing the full potential of molecules for a diverse range of devices [7–11].

At room temperature, most low-molecular weight molecules are too mobile to be stably adsorbed on a substrate unless they are part of a two-dimensional matrix [12]. This requirement invariably calls for self-assembly strategies to achieve stable integration of molecules on an atomically flat substrate. The formation of ordered structures via self-assembly depends strongly on size and symmetry of the molecule and substrate, functional groups in the molecule, matching of the lattice constants, as well as the molecule–molecule and molecule–substrate interactions. It is a priori not known if a molecule will self-assemble on any given substrate. Some of the avenues explored to ensure immobilization of the desired molecules involve functionalization of the molecule by long alkyl chains acting as anchors [13] or utilization of a molecular template [14], but they have limitations such as difficulty in the synthesis of alkyl-substituted derivatives, or unavailability of appropriate templates. Alternatively, one can use the directional nature of hydrogen bonds to induce self-assembly of the molecules containing properly positioned electronegative atoms such as N or O. As a result, the H atoms from one molecule can form non-covalent bonds with the O or N atoms of other molecules, giving rise to various stable as well as tunable molecular nanoarchitectures on solid surfaces [15–17].

Here we report on the hydrogen bonding mediated self-assembly of newly synthesized pyrazine-derived molecules at the 1-phenyloctane–HOPG interface using scanning tunneling microscopy (STM) technique under ambient conditions. The molecules belong to a new class of pyrazine/triazine-based molecules, containing two or more pyridine pendant units, and can act as a precursor to several two- and three-dimensional supramolecular architectures with tunable nanocavities [18–19]. STM images reveal that the molecular adlayer spreads over an area of several hundred square nanometers at the 1-phenyloctane–HOPG interface. Furthermore, these molecules exhibit polymorphic self-assembly on the HOPG substrate where two different molecular packing structures with nearly equal distribution are observed. Detailed analysis of the molecular packing in the two polymorphs emphasizes the crucial role of intermolecular hydrogen bonding, and molecule–substrate interactions. The observation of polymorphism may be ascribed to the nearly equal stabilization energies for the two polymorphs, or kinetic trapping effects.

## Results and Discussion

**Figure 1:**
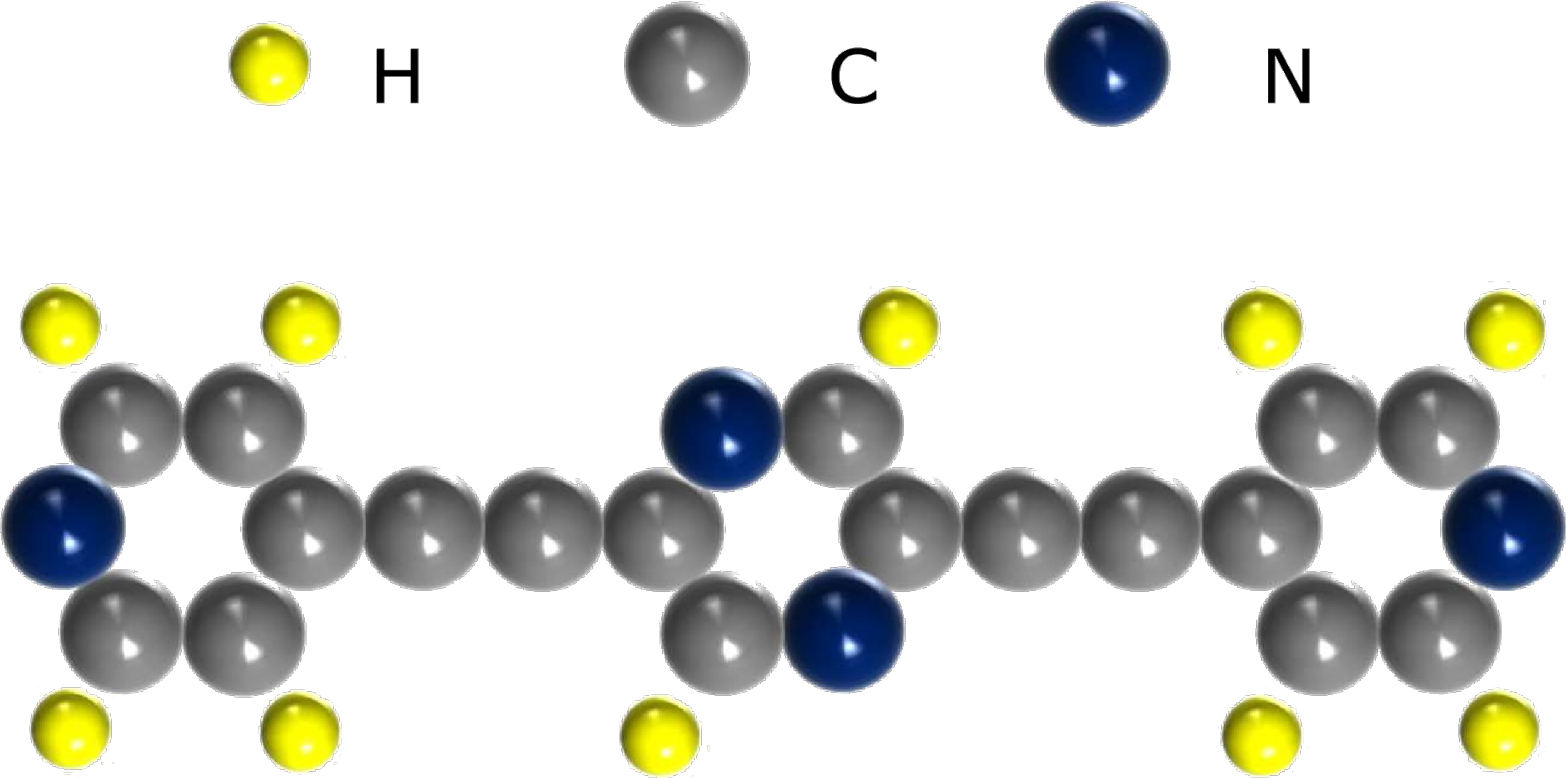
A schematic molecule structure of **1**.

2,5-Bis(pyridin-4-ylethynyl)pyrazine (**1**) reported here were newly synthesized using 2,5-dibromopyrazine as the starting material. The molecule consists of two pyridyl groups covalently connected to a central pyrazine ring at the *para*-positions, making an angle of 180° between the two binding sites, giving it a linear shape (Figure 1). Details of the synthesis of the molecule, and its characterization by means of FTIR, ^1^H and ^13^C NMR, mass spectrometry, and single crystal X-ray diffraction are provided in Supporting Information File 1, sections 1–4. Crystallographic analysis reveals that these molecules crystallize in the monoclinic space group *P*21/*c*. In a bulk crystal both the nitrogen atoms, N1 of the pyrazine ring and N2 of the pyridyl group, have intermolecular interactions with the neighboring molecules via “hydrogen bridges” (Figure S1, Supporting Information File 1). The corresponding bond parameters for such intermolecular interactions (N···H–C) are listed in Table S3 of Supporting Information File 1. The N···H distances are in the range of 0.25–0.28 nm, while the C···N distances lie between 0.34 to 0.36 nm. Based on literature reports, these interactions may be termed as “weak hydrogen bonds” since in each case a hydrogen atom bridges two atoms of relatively low electronegativity [20]. These weak H-bonding interactions, present in the crystal lattice of **1**, are shown in Figure S1b of Supporting Information File 1.

The two-dimensional self-assembly of **1** was studied at the 1-phenyloctane–HOPG interface using an ambient scanning tunneling microscope (STM). This technique allows us to investigate the surface crystallography with unprecedented spatial resolution providing information such as lattice parameters as well as the number, type, and orientation of molecules within a unit cell. In general, the STM experiments conducted under ambient conditions are prone to thermal drift and suffer from piezo creep and hysteresis. To overcome this limitation, the acquired STM images were corrected by means of split-image technique [21] in which both the adsorbate layer and the substrate are recorded with molecular and atomic resolution, respectively, in a single frame. The details of calibration and correction of STM images is given in the section 5 of Supporting Information File 1. A 5 μL droplet of a 100 μM solution of **1** in 1-phenyloctane was drop-cast on a freshly cleaved HOPG surface. The vapor pressure of 1-phenyloctane is sufficiently low to allow for stable STM imaging for several hours. Experiments performed at lower concentrations (10 and 20 μM) did not reveal stable self-assembled molecular structures on the surface. STM tips were mechanically cut from a PtIr wire and used without insulation. A bias voltage (*V*_s_) was applied to the sample with respect to the tip. No bias-voltage dependence of the STM images was observed in our experiments.

**Figure 2:**
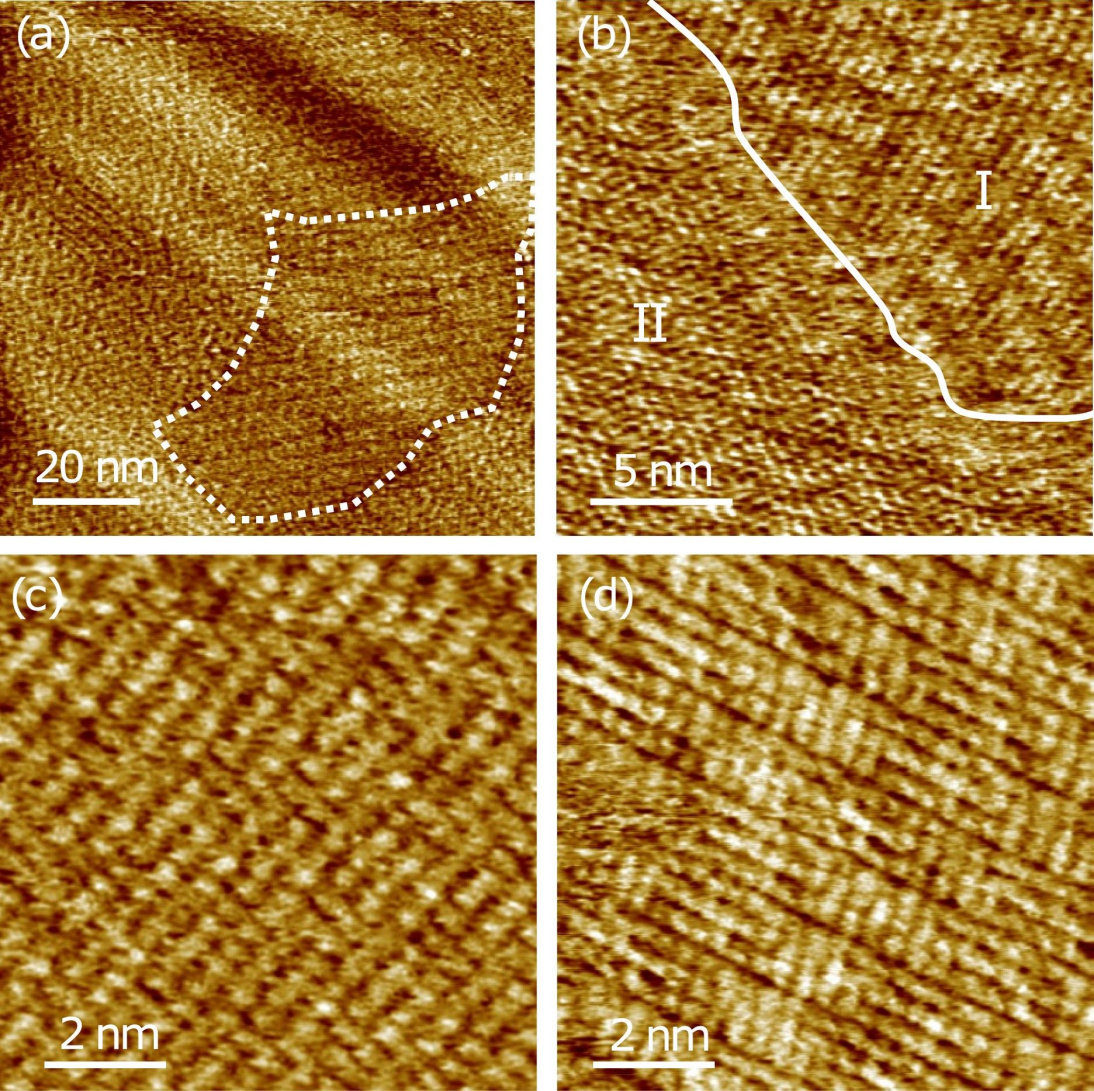
Polymorphic self-assembly of **1** at the solution–solid interface. (a) Large-scale STM image; *V*_s_ = 1.4 V, *I*_t_ = 1 nA. (b) STM image showing two polymorphs separated by a domain boundary indicated by a solid white line. *V_s_* = 0.8 V, *I*_t_ = 400 pA. (c, d) STM images with sub-molecular resolution depicting the molecular packing in the two polymorphs mentioned in the text; *V*_s_ = 0.6 V, *I*_t_ = 500 pA.

Figure 2a shows a typical large-scale STM topographic image of the 2,5-bis(pyridin-4-ylethynyl)pyrazine molecular monolayer at the 1-phenyloctane–HOPG interface. Molecules of **1** self-assemble to form ordered structures on a very large scale up to 500 nm × 500 nm. As clearly discernible in Figure 2a, these molecules undergo polymorphic self-assembly forming several domains of varying molecular packing. These domains are separated by sharp domain boundaries. One such domain boundary is indicated by dotted white line. Detailed investigation of the surface revealed two distinct polymorphs, namely, I and II, which were distributed on the surface with nearly equal probability. On a fully covered surface region, the relative area for polymorph I was determined to be (52 ± 8)%. Furthermore, several rotational domains for each packing structure were observed on the surface.

In order to reveal the precise orientation and ordering of molecules in the different polymorphs, higher-resolution STM images were obtained. Figure 2b depicts the molecular packing in a surface region containing both the observed polymorphs. The two regions are separated by a solid white line and marked as I and II. Region I corresponds to linear arrangement of the molecules leading to the formation of molecular arrays. In region II, the molecular arrangement exhibits carpet-like striations. The angle between the two length-wise orientations of the molecules in region II is measured to be approximately 70°. As depicted in the STM images with sub-molecular resolution (Figure 2c,d), each individual molecule appears as three bright protrusions corresponding to the central pyrazine ring and the two pyridyl rings. At some locations, however, especially in region II (Figure 2d), the three protrusions merge together to give a rod shaped appearance to the molecule. The length of the molecule estimated from statistical analysis of STM images is 1.4 ± 0.1 nm, which is consistent with that obtained from the detailed crystallographic analysis of the X-ray diffraction data. Moreover, the surface density of the molecules in polymorphs I and II, estimated from the STM images, is 1.18 ± 0.07 nm^−2^ and 1.12 ± 0.06 nm^−2^, respectively.

**Figure 3:**
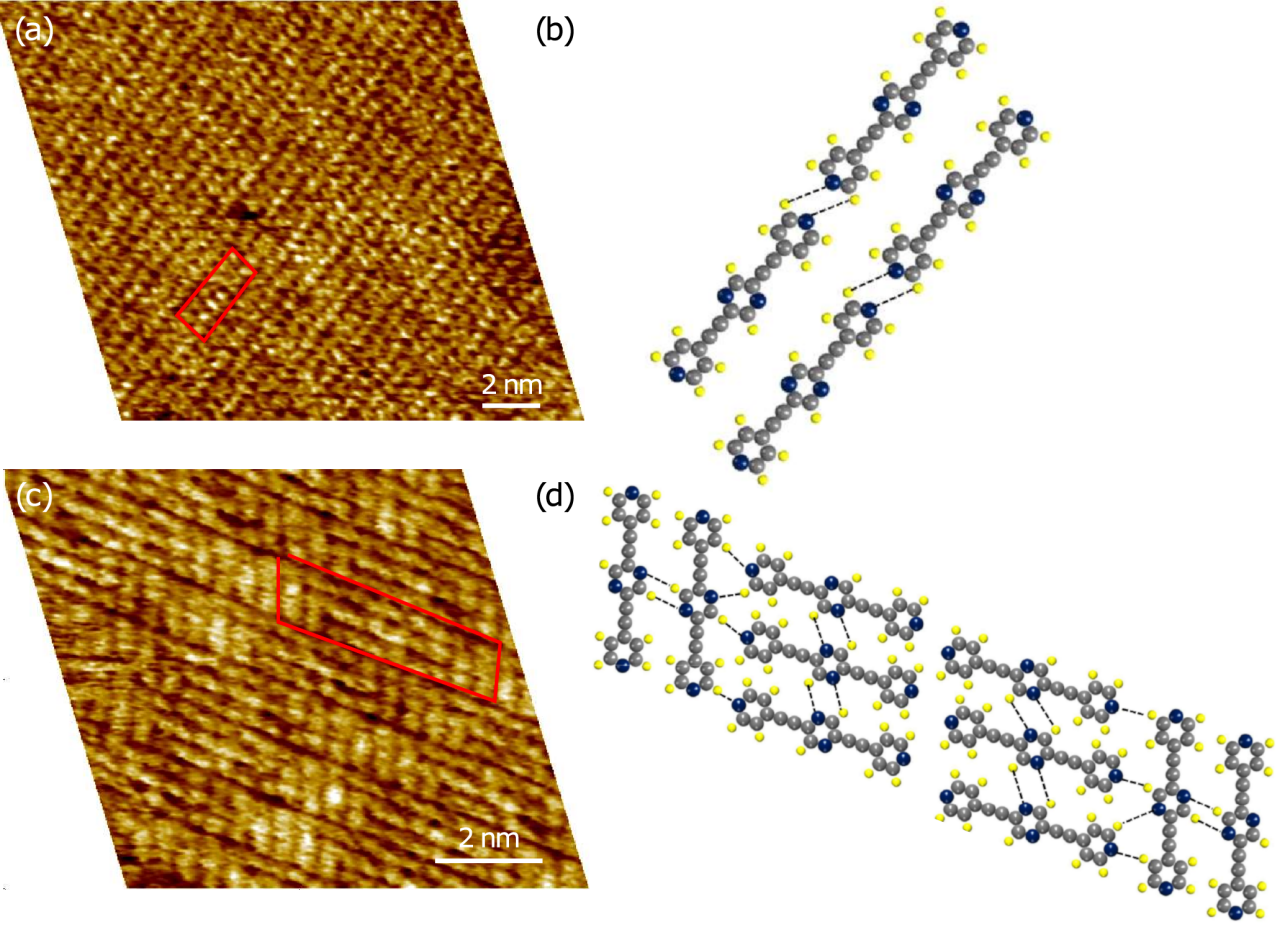
(a, c) Calibrated STM height images and (b, d) proposed molecular packing motifs of the corresponding polymorphs of **1** self-assembled at the solution–solid interface. Tunneling parameters: (a ,c) *V*_s_ = 0.6 V, *I*_t_ = 500 pA.

Figure 3 shows the high-resolution, drift-corrected, and calibrated images (Figure 3a and Figure 3c) of the two polymorphs and their proposed packing structures (Figure 3b and Figure 3d). The exact registry of the molecules on HOPG was not determined in our experiment [22]. Apparently, the adlayer formation in region I is primarily mediated by hydrogen-bonding interactions between the pyridyl groups (containing N2) of the neighboring molecules in a line. The interline separation of 0.61 ± 0.05 nm estimated from the statistical analysis of the STM images suggests van der Waals interactions, instead of hydrogen bonding, between the molecules in adjacent linear chains. Thus only the pyridyl nitrogen atoms are responsible for the molecular ordering observed in region I. On the other hand, in region II both the pyridyl nitrogen (N2) and the pyrazine nitrogen (N1) atoms apparently contribute to the intermolecular hydrogen-bonding interactions. Our proposed packing structure is depicted in Figure 3d. Here, due to the rotated orientation of the molecules, additional hydrogen-bonding sites arising from the nitrogen atoms in the pyrazine ring become available. The possibility of additional hydrogen bonds for polymorph II should be reflected in a preferential growth of such domains. However, no such preference for polymorph II was observed in our detailed STM investigation of the surface.

The absence of any distortion from the linear shape of **1** observed in our STM images clearly indicates that these molecules physisorb on the HOPG substrate and interact weakly with the substrate. In general, a strong molecule–substrate interaction often leads to a distortion of the molecular shape as reported for 1,3,5-tris(pyridin-4-ylethynyl)benzene molecules adsorbed on Cu(111) [16]. No such distortion of the molecular symmetry was observed when these molecules were adsorbed on Ag(111) substrate [15] or at the solution–solid interface [23], where the molecule–substrate interaction is weaker than that on Cu(111). A weak molecule–substrate interaction is especially important for self-assembly since the adsorbed molecules can move around on the substrate to optimize the packing structure as dictated by intermolecular interactions. For densely packed structures, both the molecule–substrate interaction as well as the intermolecular interactions are maximized. A recent density functional theory calculations indicated that for molecules with pyridine pendants on HOPG substrates, the molecule–substrate interaction energy can become comparable or even exceed the intermolecular interaction energy [24]. Although the molecular surface densities for the two polymorphs of **1**, estimated from STM images, do not differ drastically, a slightly larger value for polymorph I makes it energetically favorable. However, as mentioned earlier, no clear preference for polymorph I was detected.

One of the possible reasons for the observation of two equally distributed polymorphs could be their nearly similar stabilization energies. Thermodynamically, a monocomponent system self-assembles into an equilibrium structure that minimizes the Gibbs free energy. The entropic cost associated with adsorption on the substrate, due to the reduced degrees of freedom of molecules, is proportional to the surface density of the molecules. This loss in entropy is compensated by enthalpic contributions from the intermolecular interactions and the molecule–substrate interaction. In the present system, slightly larger surface density for polymorph I and lesser hydrogen bonding sites make it less favorable than the polymorph II considering the entropic contribution and the enthalpic contribution from the intermolecular interactions, respectively. However, this energy penalty can be compensated by greater molecule–substrate interaction owing to the larger surface density in polymorph I [24]. As a result, the stabilization energy of polymorph I may be comparable to that of the polymorph II. Elucidating the precise roles, and the relative strengths of entropic contributions, intermolecular, and molecule–substrate interactions in the system studied here, however, is only possible through detailed theoretical simulations [24–26]. For this purpose, our experimental results with sub-molecular resolution can provide valuable inputs for future theoretical calculations on related molecule–substrate systems.

Another possible reason for the observed polymorphism could be attributed to a competition between thermodynamic and kinetic effects. During the growth process, kinetic parameters such as nucleation rates and growth rates for each polymorph play a crucial role in determining the arrangement of the molecules on the surface [27]. Non-equilibrium polymorphs can form on the surface provided they have faster nucleation or growth rate than the equilibrium structure. Typically such phases are promoted by stronger intermolecular interactions [28]. Availability of additional hydrogen-bonding sites, as discussed previously, thus favors faster nucleation and growth of polymorph II. Once such structures become sufficiently large, the energy barrier for transformation to the equilibrium structure may become unsurmountable. Then the molecular system is kinetically trapped and both the equilibrium as well as the non-equilibrium polymorphs can coexist on the surface.

As a future perspective, these pyrazine-based tectons will be utilized to form modular supramolecular structures using self-assembly based on hydrogen and halogen bonds on surfaces such as graphene [29]. Both the halogen bond and the hydrogen bond have comparable strengths [30–31], and have been utilized frequently in supramolecular chemistry. Self-assembly based on halogen bonds can be realized using aryl halide molecules [30,32–33], while self-assembly based on hydrogen bonds is viable using molecules containing carboxylic groups [21,23]. Using both, halogen and hydrogen bonding, in conjunction with pyridine containing pyrazine molecules opens up intriguing possibilities to design multicomponent assemblies on solid surfaces. Such synthesis strategies using synthons than can operate side-by-side without interfering with each other are of potential importance to realize complex supramolecular structures in predictable manner.

## Conclusion

Self-assembly of newly synthesized 2,5-bis(pyridin-4-ylethynyl)pyrazine molecules at the 1-phenyloctane–HOPG interface is investigated using scanning tunneling microscopy technique under ambient conditions. It is revealed by STM images that the molecular adlayer spreads over an area of several hundred square nanometers at the 1-phenyloctane–HOPG interface and the molecules undergo polymorphic self-assembly. Detailed analysis of the molecular packing at the HOPG surface emphasizes the crucial role of intermolecular hydrogen bonding and molecule–substrate interactions in the formation of the observed polymorphs. Observation of two equally distributed polymorphs may be ascribed to their nearly equal stabilization energies, or kinetic trapping effects. These pyrazine-based molecules have shown immense potential as building blocks to several two- and three-dimensional supramolecular architectures with tunable nanocavities. A future perspective of these molecules is in the direction of developing modular supramolecular structures using the self-assembly based on hydrogen bonds and halogen bonds on surfaces such as graphene.

## Supporting Information

File 1 Additional experimental data.

File 2 Crystallographic data for **1** (deposition number CCDC 1821751).
The crystallographic data can be obtained free of charge on application to CCDC, 12 Union Road, Cambridge CB21EZ, UK; Fax: +44-1223/336-033; E-mail: deposit@ccdc.cam.ac.uk
