# Supplementary material for: Polymorphic self-assembly of pyrazine-based tectons at the solution–solid interface
Source: Beilstein J Nanotechnol. 2019 Feb 18;10:494–9. doi: 10.3762/bjnano.10.50 (PMC6404514; doi:10.3762/bjnano.10.50)
Supplement: File 1 — Additional experimental data. [file Beilstein_J_Nanotechnol-10-494-s001.pdf]

BEILSTEIN JOURNAL OF NANOTECHNOLOGY

Supporting Information

for

Polymorphic self-assembly of pyrazine-based tectons at the solution–solid interface

Achintya Jana, Puneet Mishra and Neeladri Das

Beilstein J. Nanotechnol. **2019,** *10,* 494–499. doi:10.3762/bjnano.10.50

Additional experimental data

License and Terms: This is a supporting information file under the terms of the Creative Commons Attribution License (http://creativecommons.org/licenses/by/4.0). Please note that the reuse, redistribution and reproduction in particular requires that the authors and source are credited.
The license is subject to the *Beilstein Journal of Nanotechnology* terms and conditions: (https://www.beilstein-journals.org/bjnano)

1 Synthesis and characterization of compound 1

Using 2,5-dibromopyrazine as a synthon, synthesis of 2,5-bis(pyridin-4-ylethynyl)pyrazine (**1**) was achieved in two steps (Scheme S1). A Sonogashira cross-coupling reaction of 2,5-dibromopyrazine with (trimethylsilyl)acetylene, followed by deprotection of trimethylsilyl group yielded 2,5-diethynylpyrazine. Subsequent coupling of 4-iodopyridine with 2,5-diethynylpyrazine in presence of Pd(II) catalyst and CuI co-catalyst resulted in the formation of desired organic molecule **1** in high yield. **1** was obtained as brownish solid, stable in air/moisture and has high solubility in common organic solvents. This molecule was characterized by FTIR and ^{1}H and ^{13}C NMR spectroscopy (section 2), mass spectrometry (section 3), and elemental analyses. In the FTIR spectrum of **1**, the observation of a strong band at 2228 cm^{-1} (ν C≡C str.), indicates the presence of ethynyl functional group in final products. In the ^{1}H NMR spectrum of **1** (Figure S2), the presence of sharp singlet at 8.81 ppm was assigned to the aromatic protons of the pyrazine ring in this molecule. The two sets of signals in the range of 8.70 to 7.48 ppm are attributed to the pyridyl protons in the product. The ^{13}C NMR spectrum of **1** (Figure S3) exhibited all the characteristic peaks corresponding to pyrazine, pyridine and ethynyl units in the expected region. In addition, the molecular structure of **1** was further confirmed unambiguously by single-crystal X-ray analysis (Figure S1, and section 4, Tables S1–S3).

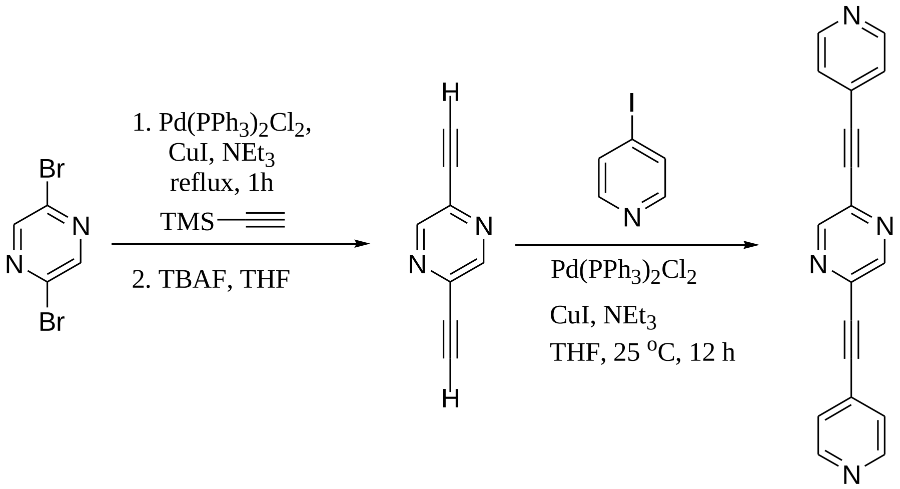

Scheme S1: Synthesis of the pyrazine-based organic molecule **1**.

X-ray analysis of 1

Figure S1 shows the results from single-crystal X-ray diffraction of compound **1** obtained by slow evaporation of its chloroform solution at ambient temperature. **1** crystallizes in the monoclinic space group *P*21/*c*. Details of the measurement and the basic crystallographic parameters are given in section 4. The molecular structure with the numbering scheme is shown in Figure S1a. Both the pendant 4-ethynylpyridine moieties are nearly coplanar with the molecular plane. The centroid of the pyrazine ring of 2,5-bis-pyridin-4-ylethynyl-pyrazine, $C_{18}H_{10}N_4$, lies on a crystallographic center of inversion. Detailed crystallographic analysis revealed that the nitrogen atoms (N1 and N2) have H-bonding interaction with the neighboring molecules (Figure S1b). The solid-state crystal structure of **1** contains two hydrogen-bond interactions. The intermolecular H-bonding geometry is listed in Table S3. The selected bond distances (Å) and bond angles (°) for **1** are shown in Table S2.

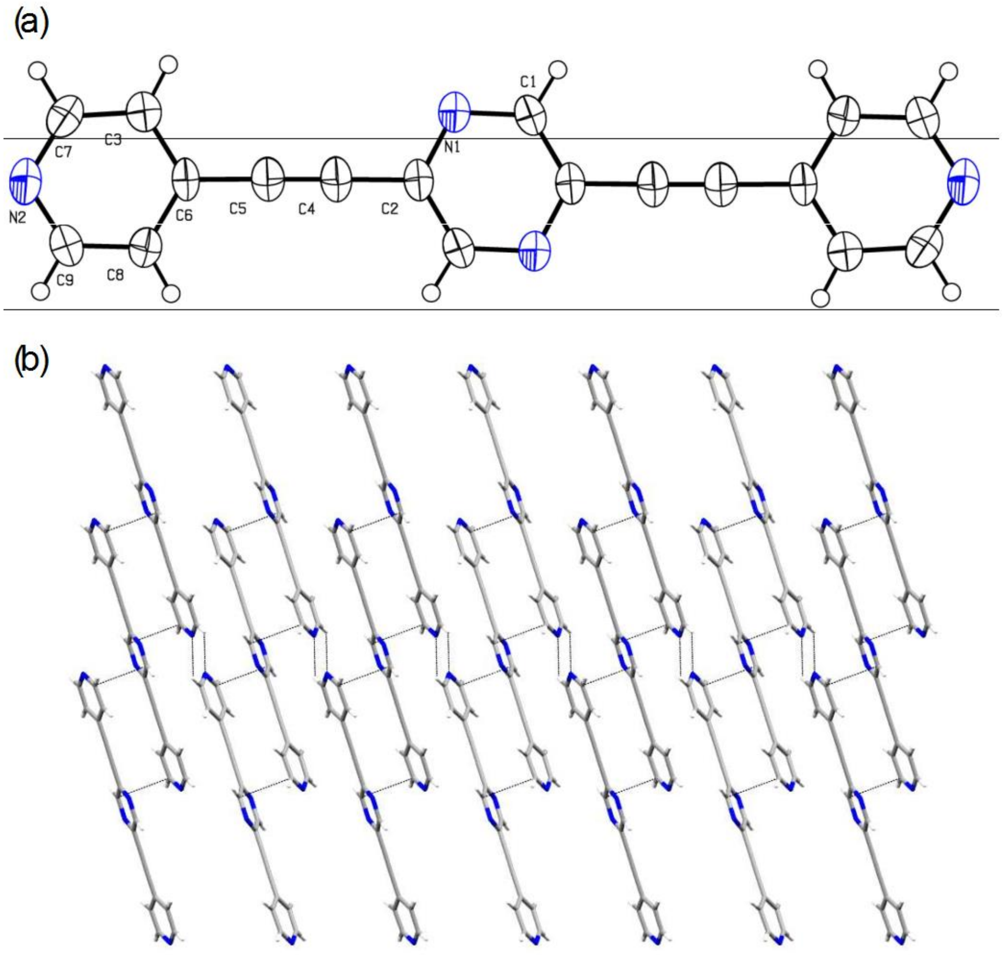

Figure S1: Characterization of 3-dimensional crystal structure of compound **1**. (a) ORTEP representation of **1**; thermal ellipsoids were drawn at the 40% probability level. (b) Hydrogen-bond interactions (fragmented bonds) in the crystal lattice of **1** as viewed along the *b*-axis.

General details

All chemicals and anhydrous solvents used in this work were purchased from commercial sources and used without further purification. Air-sensitive reactions were carried out under nitrogen atmosphere. 2,5-Diethynylpyrazine [1-3] and 1,3,5-tris(10-carboxydecyloxy) benzene (TCDB) [4] were prepared by following the reported literature procedures. Melting points were recorded using SRS EZ-Melt automated melting point apparatus by capillary methods and are uncorrected. FTIR spectra were recorded in a PerkinElmer Spectrum 400 FTIR

spectrophotometer. ^{1}H and ^{31}P NMR spectra were recorded on Bruker 400 MHz spectrometer with deuterated solvent. The elemental analyses were carried out using Elementar Micro vario Cube elemental analyzer. MS analysis was performed on Bruker Impact ESI-Q-TOF system.

Synthesis of 2,5-bis-pyridin-4-ylethynyl-pyrazine (1):

2,5-diethynylpyrazine (100 mg, 0.78 mmol), 4-iodopyridine (320 mg, 1.56 mmol), CuI (15 mg, 0.08 mmol) and bis(triphenylphosphine)palladium(II) dichloride (55 mg, 0.08 mmol) were charged in a 50 mL Schlenk flask in the glove box. Subsequently, 20 mL dry THF and freshly distilled triethylamine (0.5 mL, 3.12 mmol) were added under nitrogen. The reaction mixture was stirred overnight at room temperature. After overnight stirring, the dark brown reaction mixture was filtered through a bed of celite. The filtrate obtained was evaporated to dryness on a rotary evaporator to get crude product which was purified by column chromatography on silica gel by eluting with 30% ethyl acetate in hexane to isolate the desired product (**1**) as brownish solid.

2,5-Bis-pyridin-4-ylethynyl-pyrazine **(1)**: Yield: 0.177 g, 80%; mp 152-157 °C; ^{1}H NMR (400 MHz, $CDCl_3$): δ 8.81 (s, 2H, Ar-H), 8.70 (dd, J = 1.56 Hz, 1.56 Hz, 4H, Ar-H), 7.48 (dd, J = 1.56 Hz, 1.56 Hz, 4H, Ar-H). $^{13}C\{^{1}H\}$ NMR ($CDCl_3$, 100 MHz): δ 150.1, 147.5, 137.7, 129.3, 125.7, 91.9, 89.4. IR (ATR): 2921, 2853, 2228, 1692, 1585, 1533, 1492, 1463, 1407, 1323, 1214, 1180, 1147, 1018, 913, 814, 625 cm^{-1}. Anal. Calcd. For $C_{18}H_{10}N_4$: C, 76.58; H, 3.57; N, 19.85. Found: C, 76.70; H, 3.72; N, 19.92. HRMS (ESI, *m/z*): Calculated for $C_{18}H_{10}N_4$ ($[M+H]^+$): 283.10; Found: 283.10.

Single-crystal structure determination

A suitable single crystal of **1** was carefully selected under a polarizing microscope and mounted on a crystal mounting loop after coating with paratone oil. Single-crystal data was collected on a Bruker D8 Quest diffractometer equipped with PHOTON 100 CMOS detector, using a graphite

monochromator and Mo Kα (λ = 0.71073 Å) radiation operating at 50 kV and 30 mA. The unit cell measurement, data collection (φ and ω scan), integration, scaling and absorption corrections for the crystal was done using Bruker Apex II software [5]. The structure was solved using direct method followed by full matrix least square refinements against F^2 (all data HKLF 4 format) using SHELXTL [6]. A multiscan absorption correction, based on equivalent reflections, was applied to the data. Anisotropic refinement was used for all non-hydrogen atoms. Hydrogen atoms were placed geometrically and held in the riding model. The thermal ellipsoid plot was drawn using the program ORTEP 3 (Figure S1a), while the packing structure and the hydrogen bond interactions shown in Figure S1b were drawn using the Mercury program.

2 NMR characterization of compound 1

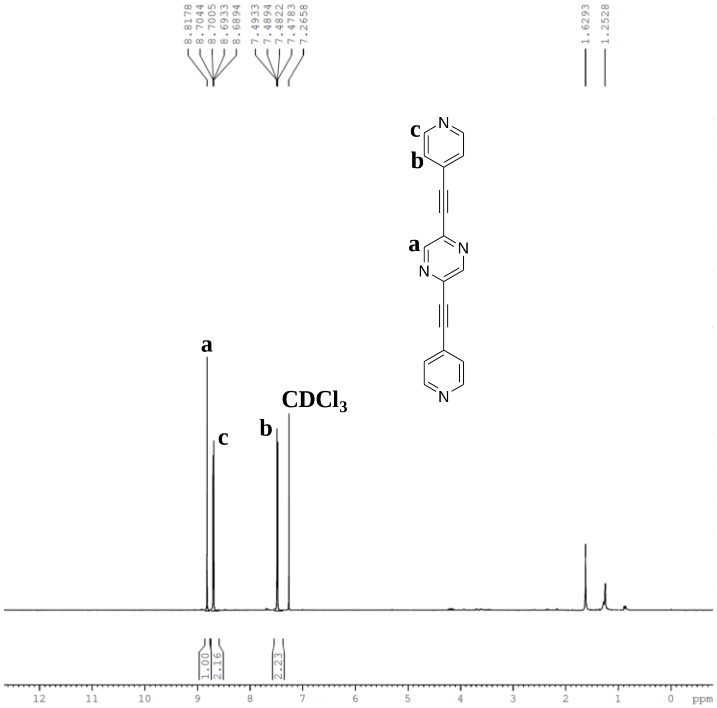

Figure S2: ^{1}H NMR spectrum of **1** (2,5-bis-pyridin-4-ylethynyl-pyrazine).

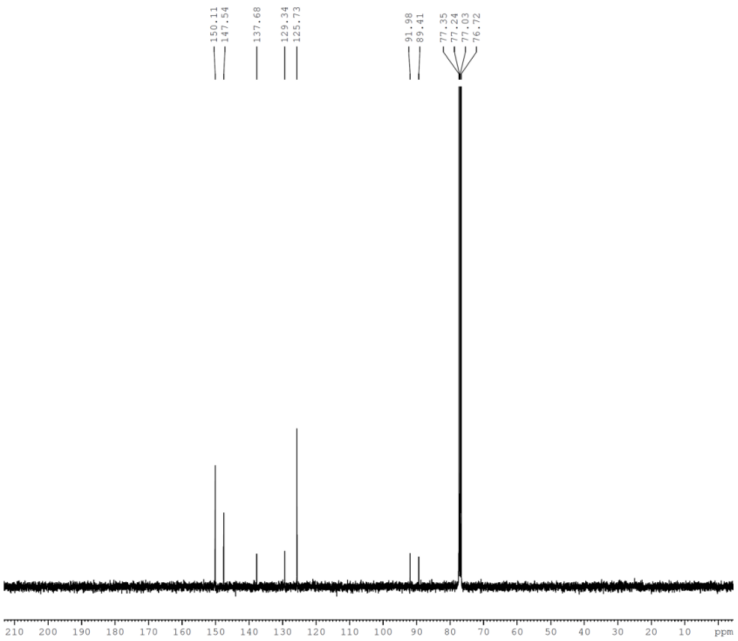

Figure S3: ^{13}C NMR spectrum of **1** (2,5-bis-pyridin-4-ylethynyl-pyrazine).

3 Electrospray ionization mass spectrometry of compound 1

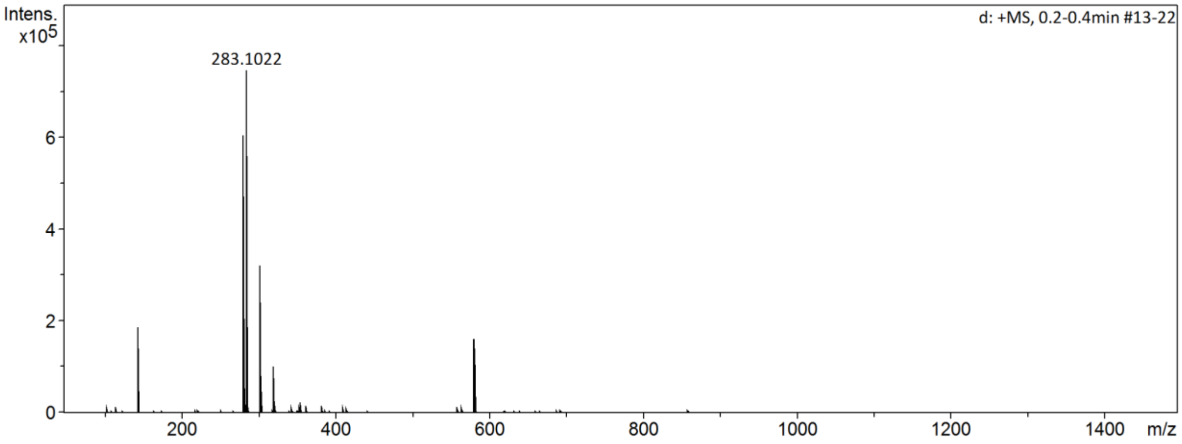

Figure S4: ESI-MS data of **1**.

4 Analysis of X-ray diffraction data of compound 1

Table S1: Crystallographic data and refinement parameters for **1.**

Identification code	**1**
formula	$C_{18}\ H_{10}\ N_4$
Mw ($gmol^{-1}$)	282.30
temp (K)	293(2)
λ (Mo Kα),(Å)	0.71073
crystal system	monoclinic
space group	P 21/c
a (Å)	8.824(9)
b (Å)	5.800(6)
c (Å)	13.935(12)
α (deg)	90
β *(deg)*	94.06(3)
γ (deg)	90
V (Å^3)	711.4(12)
Z	2
D_{calc} (g/cm^3)	1.318
μ (mm^{-1})	0.082
F(000)	292.0
Collected reflections	11723
unique reflections	1724
max and min trans	0.7457, 0.6557
Goodness-of-fit (GOF) on F^2	1.011
R1 [$I > 2\sigma(I)$][a]	0.0625
wR2 (all data)[b]	0.1425
CCDC Number	1821751

[a]$R_1 = \sum \lvert \lvert F_o \rvert - \lvert F_c \rvert \rvert / \sum \lvert F_o \rvert$; [b]$wR_2 = \{\sum [w(F_o{}^2 - F_c{}^2)^2] / \sum [w(F_o{}^2)^2]\}^{1/2}$

Table S2: Selected bond distances (Å) and bond angles (°) for **1**.

bond lengths (Å)			
N1— C1	1.323(3)	C3—C6	1.378(3)
N1—C2	1.334(3)	C4—C5	1.187(3)
N2—C9	1.316(3)	C5—C6	1.433(3)
N2—C7	1.319(3)	C6—C8	1.374(3)
C2—C4	1.435(3)	C8—C9	1.371(3)
C3—C7	1.376(3)		
bond angles (deg)			
C1 - N1 - C2	115.45(19)	C3 - C6 - C8	117.3(2)
C7 - N2 - C9	122.9(2)	C5 - C6 - C8	121.0(2)
N1 - C2 - C4	117.3(2)	N2 - C7 - C3	124.8(2)
C6 - C3 - C7	118.5(2)	C6 - C8 - C9	119.2(2)
C2 - C4 - C5	179.0(2)	N2 - C9 - C8	124.4(2)
C4 - C5 - C6	179.0(2)		
C3 - C6 - C5	121.7(2)		

Table S3: Hydrogen bond parameters found in **1**.

**D—H···A**	**D—H (Å)**	**H···A (Å)**	**D···A (Å)**	**<D-H-A (°)**	**Symmetry**
C7 — H7···N1	1.080	2.723	3.602	138.31	-x,-y+1,-z+1
C9 — H9···N2	1.080	2.581	3.432	135.13	-x-1,+y-1/2,-z+1/2

5 Calibration of the scanning tunneling microscope

In general, the STM experiments conducted under ambient conditions are prone to thermal drift and suffer from piezo creep and hysteresis. To overcome this limitation, the acquired STM images were corrected by means of split-image technique [7] where both the adsorbate layer and the substrate are recorded with molecular and atomic resolution, respectively, in a single frame. For the dipyridyl-4-ylethynyl pyrazine molecules, such split image could not be acquired. We instead used a well-known molecule 1,3,5-tris(10-carboxydecyloxy) benzene (TCDB) to calibrate the STM instrument. Prior to our experiment on pyrazine-based tectons, a monolayer of

TCDB molecules at the 1-phenyloctane–HOPG interface was prepared. A composite STM image was obtained for this surface is shown in Figure S5a. The corresponding 2D fast Fourier transform (FFT) (Figure S5b) of the HOPG region in the image clearly reveals six Fourier peaks. However, the six peaks form an irregular hexagon and do not lie on a circle, as expected for HOPG lattice, due to the thermal drift, piezo creep and other calibration issues that are unavoidable under ambient conditions. The distortion parameters were estimated following the recipe prescribed in [8] and the image was restored using the SPIP software from Image Meterology. The restored image of the surface is shown in Figure S6a. The Fourier peaks in the FFT of the restored image of the HOPG region now lie on a circle and form a regular hexagon (Figure S6b). The parameters estimated above were used to restore the STM images of the TCDB monolayers, yielding unit cell size consistent with earlier experimental and theoretical results [9]. Same distortion parameters were utilized to restore the molecularly resolved STM images of pyrazine-derived molecules presented here.

In order to ensure that the parameters estimated from our experiment on TCDB molecules provide a reasonably correct estimate of the drift, both the high-resolution STM images for the pyrazine-derived molecules and the composite STM images for TCDB molecules was acquired after stabilizing the tip for 3–4 h at the same location. In addition, the imaging parameters, namely, scan size, scan speed, and scan angle, were the same for both cases.

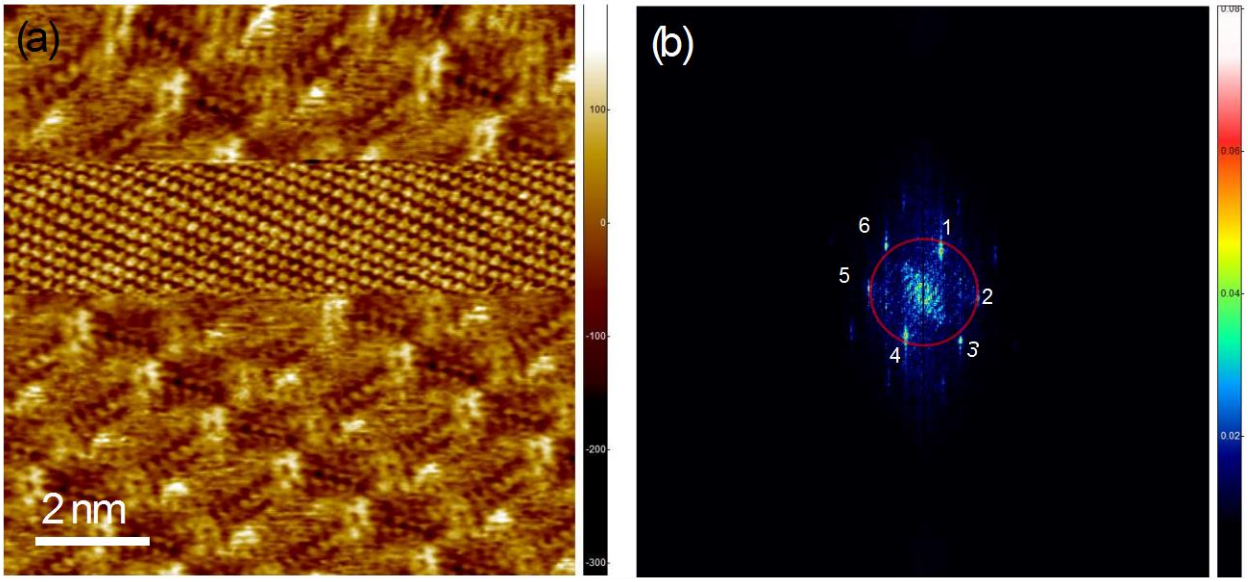

Figure S5: A composite STM image showing both the atomically resolved underlying HOPG lattice and molecular lattice of the TCDB molecules at the 1-phenyloctane–HOPG interface.

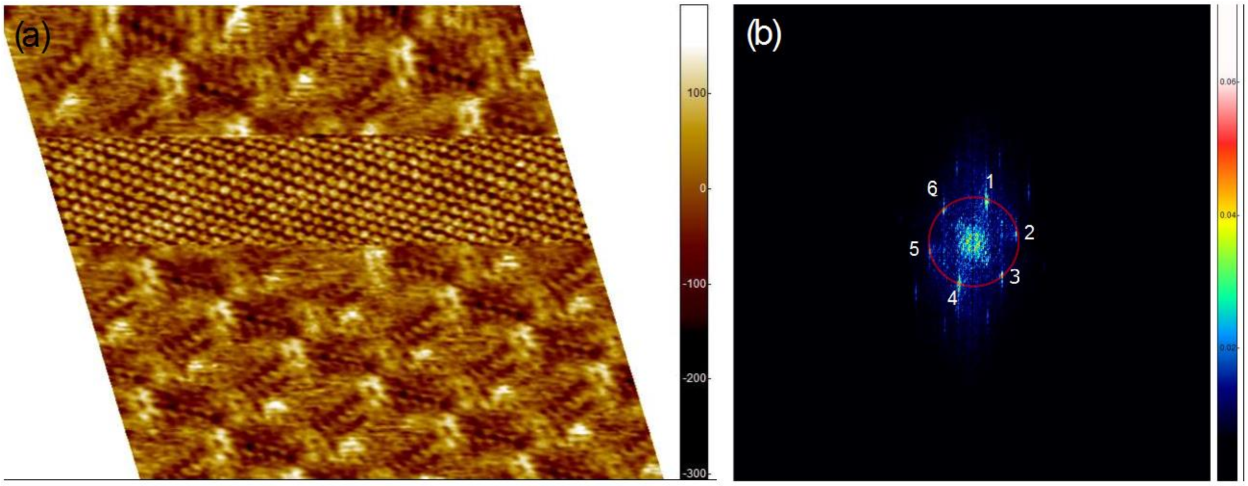

Figure S6: (a) The restored composite STM image after calibration. (b) FFT of the restored image depicting that the reciprocal lattice peaks are now located on a circle.

References

1. Pieterse, K.; Lauritsen, A.; Schenning, A. P. H. J.; Vekemans, J. A. J. M.; Meijer, E. W. *Chem. – Eur. J.* **2003,** *9,* 5597–5604. doi:10.1002/chem.200305073
2. Bhowmick, S.; Jana, A.; Singh, K.; Gupta, P.; Gangrade, A.; Mandal, B. B.; Das, N. *Inorg. Chem.* **2018,** *57,* 3615–3625. doi:10.1021/acs.inorgchem.7b01561
3. Bhowmick, S.; Chakraborty, S.; Marri, S. R.; Behera, J. N.; Das, N. *RSC Adv.* **2016,** *6,* 8992–9001. doi:10.1039/C5RA21484J
4. Lu, J.; Lei, S. B.; Zeng, Q. D.; Kang, S. Z.; Wang, C.; Wan, L. J.; Bai, C. L. *J. Phys. Chem. B* **2004,** *108,* 5161–5165. doi:10.1021/jp037508j
5. Apex2, Version 2 User Manual, M86-E03078, Bruker Analytical X-ray Systems: Madison, WI, USA, 2010.
6. Sheldrick, G. M. *SHELXTL Program for the Solution of Crystal of Structures*, University of Göttingen: Göttingen, Germany, 1993.
7. Lakinger, M.; Hekl, W. M. *Langmuir* **2009,** *25,* 11307–11321. doi:10.1021/la900785f
8. Jørgensen, J. F.; Madsen, L. L.; Garnaes, J.; Carneiro, K.; Shaumburg, K. *J. Vac. Sci. Technol., B: Microelectron. Nanometer Struct.–Process., Meas., Phenom.* **1994,** *12,* 1698–1701. doi:10.1116/1.587266
9. Kong, X.-H.; Deng, K.; Yang, Y.-L.; Zeng, Q.-D.; Wang, C. *J. Phys. Chem. C* **2007,** *111,* 17382. doi:10.1021/jp074682p
